# Design and Test of an Integrated Random Number Generator with All-Digital Entropy Source

**DOI:** 10.3390/e24020139

**Published:** 2022-01-18

**Authors:** Luca Crocetti, Stefano Di Matteo, Pietro Nannipieri, Luca Fanucci, Sergio Saponara

**Affiliations:** Department of Information Engineering, University of Pisa, Via G. Caruso, 16, 56122 Pisa, Italy; luca.crocetti@phd.unipi.it (L.C.); stefano.dimatteo@phd.unipi.it (S.D.M.); luca.fanucci@unipi.it (L.F.); sergio.saponara@unipi.it (S.S.)

**Keywords:** random number generator, FPGA, entropy, ASIC, EPI

## Abstract

In the cybersecurity field, the generation of random numbers is extremely important because they are employed in different applications such as the generation/derivation of cryptographic keys, nonces, and initialization vectors. The more unpredictable the random sequence, the higher its quality and the lower the probability of recovering the value of those random numbers for an adversary. Cryptographically Secure Pseudo-Random Number Generators (CSPRNGs) are random number generators (RNGs) with specific properties and whose output sequence has such a degree of randomness that it cannot be distinguished from an ideal random sequence. In this work, we designed an all-digital RNG, which includes a Deterministic Random Bit Generator (DRBG) that meets the security requirements for cryptographic applications as CSPRNG, plus an entropy source that showed high portability and a high level of entropy. The proposed design has been intensively tested against both NIST and BSI suites to assess its entropy and randomness, and it is ready to be integrated into the European Processor Initiative (EPI) chip.

## 1. Introduction

The generation of random bits (or numbers) represents one of the fundamental and most significant aspects concerning cybersecurity, because they are employed to generate and/or derive cryptographic keys, one-time passwords, initialization vectors (IVs) for some cryptographic algorithms, and more in general non-repeating values (as the nonces and others). From here, the requirement of having good-quality random numbers becomes a mandatory feature for those security modules aimed to provide and strengthen the security level of a system or an application. With good-quality random numbers, they indicated those sequences of bits generated unpredictably, for which the higher the unpredictability, the higher the quality (or the security strength). Indeed, if the method used to produce such streams of bits was predictable, also only in part, this would expose an entire system to severe security threats. For example, it can be assumed that a cybersecurity device offers confidentiality protection of data by using a symmetric-key encryption/decryption scheme. The key employed within the encryption and decryption functions must be protected as well, because if an attacker was able to discover or to guess such a key, then it would have access to the content of the communication, compromising the privacy between the entities. Random numbers could be used to generate or refresh such keys, or also as the initial value of a packet number that is integrated into the communication to protect against replay attacks. Anyway, the higher the probability to retrieve the value of those random numbers, the higher the probability for an adversary to obtain such data.

In addition to next-generation technologies, such as quantum-based random number generation [1], there are essentially two methods to generate random bits exploitable in digital circuits. One strategy consists of producing bits non-deterministically, for which the value of every output bit is based on a physical process that is intrinsically unpredictable: this class of Random Bit Generators (RBGs) is commonly known as Non-deterministic Random Bit Generator (NRBG). The other approach consists of computing random bits using a deterministic algorithm, and accordingly, this class of RBGs is known as Deterministic Random Bit Generator (DRBG). Such a class of RBGs produces sequences of bits from an initial value that is denoted as seed, and it is required to be the output of a source of randomness. As a result of the deterministic nature of the process, a DRBG is said to produce pseudo-random bits rather than random bits. Moreover, the seed used to initialize the DRBG can be compared to a cipher key that must be kept secret; otherwise, its knowledge can allow an attacker to disclose the future values of pseudo-random sequence, as it is generated in a deterministic way. If on one hand, the terms DRBG and NRBG refer to the construction techniques and properties of RBGs, on the other one, their implementations are usually denoted as Pseudo-Random Number Generators (PRNGs) and True Random Number Generators (TRNGs), respectively, because they are typically used to generate random values with a width greater than 1 bit (notably a number). In this field, the adoption of modules named Cryptographically Secure Pseudo-Random Number Generators (CSPRNGs) also acquired relevance, which are PRNG with specific properties regulated by standards, and whose output sequence has a degree of randomness such that it cannot be distinguished from an ideal random sequence within certain limits; for this reason, they can be employed in cryptographic applications. A CSPRNG can be implemented both integrating a source of randomness or a TRNG by itself, thus beginning an only output module that does not require any input (i.e., the seed), and without integrating such source of randomness: in such case, the CSPRNG module is provided also with an input interface for seeds that must respect specific and stringent rules specified by the corresponding standards.

This article focuses on the design and test of an all-digital hardware accelerator for random number generation, NIST-compliant at entropy level, with sustained throughput and technology independence. The complete hardware accelerator is to be exploited within the 7 nm European Processor Initiative [2] ASIC, together with other hardware accelerators that will rely on it (AES [3], ECC [4], SHA [5]). The paper is organized as follows. In Section 2, the architecture of the proposed RNG is explained. In particular, this section covers also the aspects related to the design of both the entropy source and the DRBG modules as well as the synthesis process on the target technology; Section 3 focuses on the achieved results in terms of complexity, entropy, and randomness of the proposed design; Section 4 compares the obtained results with the State of the Art, and finally, Section 5 points out the conclusions for this work.

## 2. Design of the Random Number Generator

### 2.1. RNG Engine

The RNG engine implements a CSPRNG to provide random sequences of bits (or numbers) with an entropy level that can be considered sufficient for cryptographic applications requiring a high level of security (or security strength).

**Figure 1:**
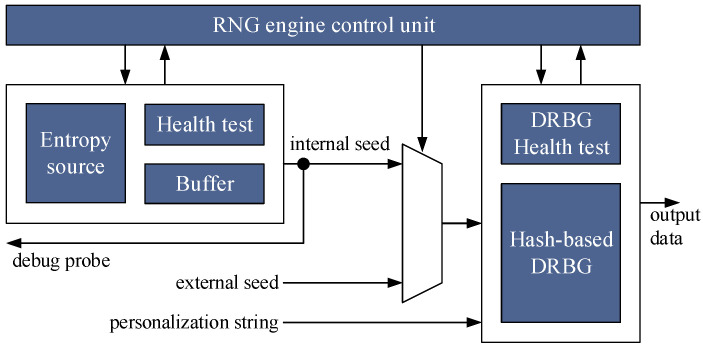
Internal architecture of RNG engine.

The internal architecture of the RNG engine is illustrated in Figure 1, and it mainly consists of the integration of an entropy source module and a hash-based DRBG module, which are respectively described in [6,7,8]. The former is responsible for the generation of (internal) seeds that are used to initialize the internal state of the DRBG and that are built byte by byte, exploiting the Buffer unit to collect them, while the latter produces sequences of random words that constitute the output data of the whole RNG engine. Both modules are equipped with their own dedicated health test mechanism that continuously evaluates the randomness of the generated bitstreams, and both are managed by a control unit that regulates their usage and interaction, which is also based on the response of the health test. Moreover, the DRBG module accepts as input also personalization string to further increase the degree of randomness of output sequences, according to the specifications of [9,10], and it also seeds from the external: this choice was guided by the fact that the entropy level of seeds generated by the entropy source module is strongly dependent on the technology used for its implementation, and it was not possible to evaluate its characteristics in advance, using some statistical models. Thus, it was foreseen to permit the usage of seeds from external to improve the robustness of the RNG engine and prevent the impossibility of using at least the DRBG part of the CSPRNG, in case the integrated entropy source module is not able to generate seeds featuring the required amount of entropy. This solution allows also to continue using the DRBG mechanism of the RNG engine in case the entropy module shows some flaws during its operation, and that is signaled by the corresponding Health test unit. Finally, an additional and dedicated output line has been included in the RNG engine to access and acquire the raw data from the entropy source module and conduct tests to assess what the entropy level can provide: such an output line corresponds to the signal debug probe in Figure 1. With respect to the previous works published in [6,7,8], this paper presents the whole design of the RNG engine and introduces some architectural features for improving its robustness such as the input ports for external seed and personalization string, the output port for the entropy source assessment, and the health test modules for both the entropy source and the DRBG module. In addition, with respect to the work in [6], this work shows the complete evaluation procedure for the whole RNG engine and demonstrates the portability of the entropy source in different FPGA technologies.

### 2.2. Entropy and Design of the All-Digital Entropy Source

Concerning the entropy source module, its development was guided by the purpose of implementing a digital design featuring the highest level of entropy, and that was portable on a variety of technologies, exploiting the properties of HDL digital designs. As shown in [6], such design activity starts with the analysis (and the comparison) of the available solutions, which all rely on the usage of ROs for the generation of entropy. A RO essentially consists of a chain of combinational logic gates and cells that closes on itself, forming a circle, or a loop, in which the output of the last element corresponds also to the input of the first element of the chain. The output of the last element of the chain is used also as the output of the RO, and it oscillates between two voltage levels corresponding to the logic states *low* (or 0) and *high* (or 1). The characteristics of the oscillation depend on the topology of the combinational loop and the logic cells used to build it. Based on this, different approaches can be used to exploit some intrinsic physical phenomena such as temperature, voltage, or noise fluctuations to randomize the oscillation and thus generate a random sequence of 0 s and 1 s by sampling the output of the RO. The most diffused and main solutions are Transition Effect Ring Oscillator (TERO), originally proposed in [11], Metastable Ring Oscillator (Meta-RO), proposed for the first time in [12], Fibonacci Ring Oscillator (FiRO), and Galois Ring Oscillator (GaRO), illustrated in [13] and Fibonacci-Galois Ring Oscillator (FiGaRO), which is a combination of the last two previous candidates, and it is described, for example, in [14].

FiRO derives from its namesake LFSR architecture, replacing all D flip-flops with inverters. Randomness is introduced by the dependence of the inverter delay on temperature and supply voltage: as these parameters vary, due to both noise and environmental changes, the ring evolves with different output patterns, resulting in a chaotic and unpredictable signal. Further randomness can be acquired during the sampling phase with the violation of setup and hold times. Similarly, GaRO is derived from the Galois LFSR structure, again replacing all D flip-flops with inverters. The same considerations made with FiRO on the stochastic evolution of this circuit apply. The structure formed by XORing FiRO and GaRO is called FiGaRO structure. For more details, please refer to [6], where architectures of the mentioned oscillators are explained in detail. The selection of the FiGaRO oscillator has been made after the experimental campaign performed in [6], where it was demonstrated to be the best among all the other presented oscillators in terms of entropy for an FPGA device.

Table 1 shows the main outcome of the comparative analysis in [6], for which the FiGaRO approach was selected, because of the advantages offered by its features, including its independence to the placement of design elements and its enhanced robustness to the implementation of single FiRO or GaRO.

**Figure 2:**
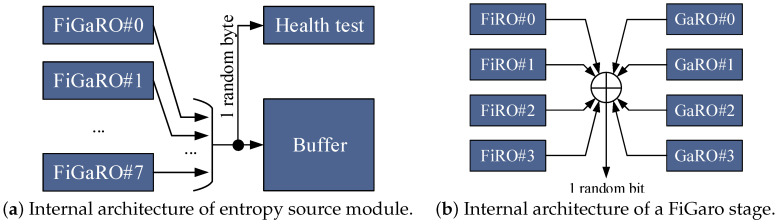
Internal architecture of entropy source module of RNG engine (**a**), and of FiGaRO stages composing it (**b**).

According to these considerations, our proposed entropy source module has been implemented by instancing eight parallel FiGaRO stages, to generate a random byte made of eight independent bits, each one from a different FiGaRO stage. The architecture of this module is illustrated in Figure 2a, while Figure 2b shows the internal structure of a FiGaRO stage, which counts four FiROs and four GaROs units mixed through the XOR gate, as this solution showed in [6] to provide an entropy per bit of 0.995, independently from the placement and the value of the sampling frequency.

### 2.3. Design of the Deterministic Random Bit Generator Module

**Figure 3:**
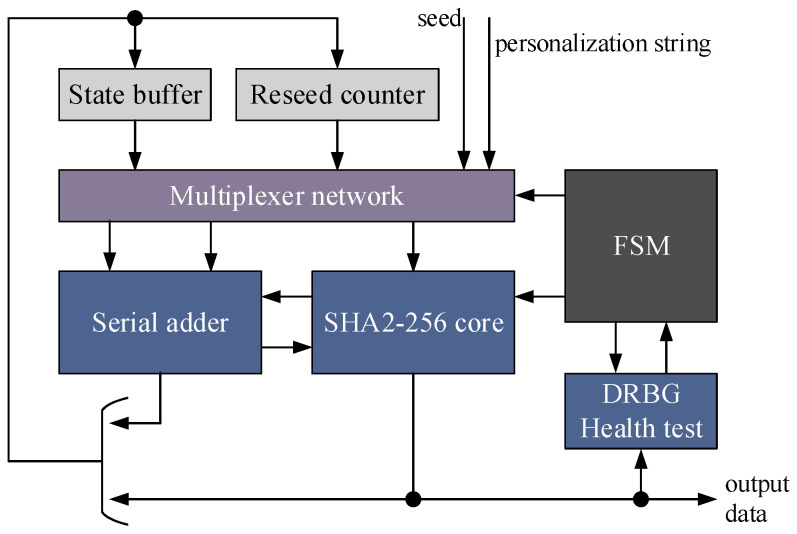
Internal architecture of DRBG module of RNG engine.

Figure 3 shows the internal architecture of the DRBG module, which relies on a SHA2-256 core and integrates also a buffer containing the current state and a reseed counter. The output of the SHA2-256 [5] core is used to generate the output stream of the DRBG; therefore, it is constituted by random words of 256 bits, corresponding to the digest of the hashing unit. The reseed counter is in charge to signal when a new seed is required in input to the module, to refresh the entropy level of the output random stream, and to avoid it decreasing too much.

The choice of a hash-based mechanism for the implementation of the DRBG module can be found in the work presented in the publications [7,8], in which they have investigated the structures and the characteristics in terms of security and performance of the implementation approach specified by the NIST standard for the construction of cryptographically secure deterministic RBGs, i.e., [9]. We propose and specify the following three main architectures:

- CTR-DRBG, which relies on the CTR mode of operation of block ciphers;
- Hash-DRBG (ref. [9] specifies also the usage SHA1 function for the Hash-DRBG class; anyway, it has not been included in the analysis presented in [8] because it offers a lower security strength to the SHA2 counterpart, and it is considered outdated), which relies on the SHA2 functions;
- HMAC-DRBG, which relies on the HMAC scheme of hash algorithms.

All these approaches can provide a security strength up to 256 bits, under specific design rules, and show different characteristics at an architectural level, which reflects also on performance. Although the CTR-DRBG solution permits implementing more efficient PRNGs both for the relative metric of throughput per area and for the absolute metrics of throughput (the highest one) and consumption of logic resources (the lowest one), it has been discarded, as [18] expresses about the effective capability of this mechanism to reach maximum security strength: its authors claim that the usage of DRBGs based on block ciphers should be avoided since the pseudo-random permutation inside each AES [3] round outputs a sequence that is distinguishable from a random source, while the DRBGs based on hash functions satisfy the security requirements. On the other hand, the DRBGs based on HMAC can be interpreted as a more complex version of the ones relying on hash functions, because they exploit the same underlying hash algorithm to which the HMAC scheme adds resources, data (the key), and operations (hence latency), therefore leading to a less efficient solution and introducing the issues related to the establishment of cryptographic keys. After these considerations, the most convenient choice lies in the Hash-DRBG approach. In this regard, it is useful to recall that SHA2-224 and SHA2-256 are in essence the same algorithm that only provides outputs of different sizes, hence requiring the same amount of resources and showing the same critical path (i.e., the same maximum frequency) but outputting a different amount of data. For this reason, the function with the highest data size (SHA2-256) must be preferred, because it offers a higher throughput at the same area cost. Similarly, such evaluation applies also to the SHA2-384 and SHA2-512 functions, for which the SHA2-512 algorithm is shown to be more efficient than the SHA2-384 one. Proceeding with a comparison of solutions based on SHA2-256 and SHA2-512 routines, no matter the implementation strategy, the function for the generation of 512-bit digests requires almost double the resources requested by the 256-bit counterpart because it operates on vectors of double size: input blocks of 1024 bits, rather than 512 bits, and internal data path of 64 bits, rather than 32 bits. On the timing performance side, the two solutions can support approximately the same clock frequency ($f_{clk}$) but with different latencies: 64 clock cycles for the SHA2-256 case and 80 clock cycles for the SHA2-512 case. Including also the length of the output digests, their throughput (At confirmation of the considerations on the HMAC-DRBGs approach, the corresponding throughput of HMAC version of the same 256-bit and 512-bit PRNGs can be expressed, respectively, such as $1\cdot f_{clk}$ bit/s and $1.6\cdot f_{clk}$ bit/s) can be expressed, respectively, like $4\cdot f_{clk}$ bit/s and $6.4\cdot f_{clk}$ bit/s. If including also the area cost, a heuristic efficiency factor can be derived by computing from the ratio between the throughput and the resources consumption as:

- $4\cdot f_{clk}\;\frac{\mathrm{bit}/s}{\mathrm{area}}$, for the SHA2-256 case;
- $3.2\cdot f_{clk}\;\frac{\mathrm{bit}/s}{\mathrm{area}}$, for the SHA2-512 case.

Therefore, in conclusion, the implementation of a Hash-DRBG module based on the SHA2-256 function should be preferred to ensure a compact and efficient solution, and that was the choice for the DRBG block in Figure 1. According to NIST specifications [9], the length of seed required by this deterministic RBG is 440 bits and the minimum level of entropy is 256 bits (while the maximum is of $2^{35}$ bits). The input seed has been extended to 512 bits both for simplicity of usage, because the SHA2 function takes as input data blocks of 512 bits, and for relaxing the constraints on the entropy degree (This minimum level of entropy per bit is calculated as the ratio between the minimum required entropy, i.e., 256 bits, and the length in bits of input seed after extension, i.e., 512 bits; if using the length of the bit of input seed specified by the NIST in [9], i.e., 440 bits, the corresponding minimum level of entropy per bit is 256/440=0.582 bits, which constitutes a more stringent requirement for the seed properties to the previous case) of input seed that must be at least of 256/512=0.5 bits of entropy per bit.

### 2.4. Synthesis Design

**Figure 4:**
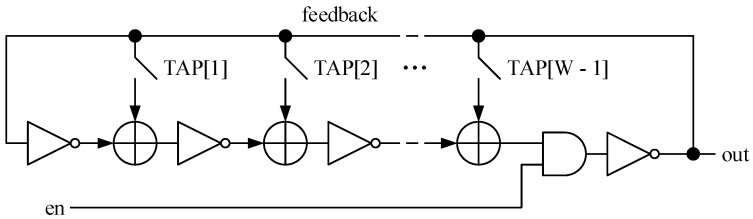
Schematic of a GaRO with enable (en signal) and *W* inverting elements.

The synthesis process of the RNG engine was performed using the Electronic Design Automation (EDA) tools Design Compiler by Synopsys [19] and Vivado by Xilinx [20], targeting, respectively, standard-cell and FPGA technologies. Concerning this step, some issues are required to be addressed for guiding the logic synthesis to generate a netlist corresponding to the expected outcome. The main issue faced during the synthesis concerned the ring oscillators (FiROs and GaROs) that are essentially a looped chain of *NOT* and *XOR* gates, as shown by Figure 4 for a GaRO of W inverting elements. The inverting elements are the *NOT* gate and the global enable signal of the circuit is *en*, while the TAP[i] elements are the coefficient of the polynomial associated to the feedback net: if TAP[i] = 1, the corresponding connection is a short-circuit; hence, the corresponding XOR gate is instantiated within the circuit; otherwise, (TAP[i] = 0) the corresponding connection is an open circuit, and the associate *XOR* gate is not instantiated.

**Figure 5:**
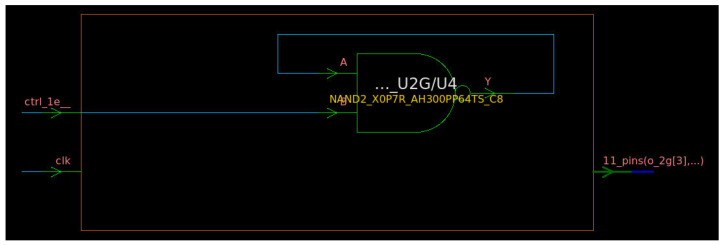
Typical outcome of the synthesis of a GaRO. This schematic has been extracted by using the Synopsys Design Compiler tool.

When generating the corresponding netlist with the library cells of the target technology, the synthesis engine performs several optimizations that can include also the modification of some parts of the circuit described in HDL but without modifying its logical functionality. This can happen also to the ring oscillator in Figure 4, and indeed, this phenomenon was observed during the synthesis of the RNG engine. Figure 5 shows the typical outcome of the logic synthesis when trying to synthesize a circuit similar to the one reported in Figure 4.

As illustrated in Figure 5, the chain of *NOT* and *XOR* gates forming the ring oscillator loop can be replaced by a single *NAND* gate by the synthesis process, because they are logically equivalent. Indeed, it can be easily proved by building the truth table associated with the *NAND* gate in Figure 5, by referring to its input ports, i.e., *A* and *B*, and its output port, i.e., *Y*.

**Figure 6:**
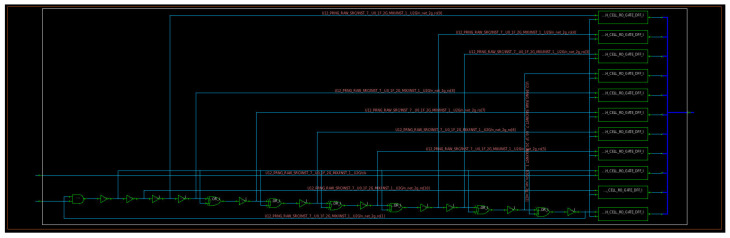
Outcome of GaRO synthesis with proper constraints. This schematic has been extracted by using the Synopsys Design Compiler tool and refers to the synthesis of the same RTL code used also for the synthesis of the circuit represented in Figure 5 but including dedicated synthesis constraints.

Combining the information in Table 2 with the schematic in Figure 5, it derives that when input *B* is 0, the signal on port *Y* is forced to the high logic level (1), whatever the initial value of port *A*, and it remains on that logic level, because due to the feedback wire, the value of *Y* is applied also to port *A*, and when B=0 and A=1, Y=1 (Table 2). In other words, when the module in Figure 5 is disabled (i.e., *B* which is connected and corresponds to the module enable signal, ctrl_1e__, is zero), the loop does not oscillate and the output of the module (*Y*) is stable to the logic value 1. On the other hand, when the module in Figure 5 is enabled (i.e., ctrl_1e__ =B=1), the oscillation is triggered and the output *Y* starts to bounce between the logic states 0 and 1. Assuming A=1 for instance, *Y* becomes 0 (case B=1 and A=0 in Table 2), and this value is transmitted to port *A*, which moves to the low logic value, too. Now that A=0, *Y* moves to the logic value 1 (case B=1 and A=0 in Table 2); thus, also, *A* assumes again the high logic state, and the cycle starts again from the beginning, being continuously repeated until B=1. To resolve this issue, specific constraints have been specified relying on the set_dont_touch attribute that was integrated within the Synopsys Design Constraint (SDC) file used in the synthesis flow, to force the synthesis engine to generate a netlist as close as possible to the one in Figure 4 and described accordingly in the RTL code of the RNG. An example of the results of this strategy is illustrated in Figure 6.

## 3. Results

### 3.1. Results on FPGA and 7 nm Standard Cell Technologies

In the case of FPGA, the full implementation flow was performed, i.e., logic synthesis, placement, and routing steps. The target device for this flow was a Xilinx Virtex UltraScale+ HBM FPGA VU37P [21] (device XCVU37P-L2FSVH2892EES9837) that is manufactured using a 16 nm low-power FinFET+ process technology from TSMC. The main programmable logic element of this device is named Configurable Logic Block (CLB), and it integrates several logic resources, including Look-Up Tables (LUTs) (referred to as CLB LUTs), flip-flops (referred to as CLB Registers), and other logic blocks; specifically, that FPGA counts 162,960 CLB, and each one is provided with eight LUTs (for a total of 1,303,680 CLB LUTs) and 16 flip-flops (for a total of 2,607,360 CLB Registers or bits). Moreover, such an FPGA is equipped also with other embedded hardware resources as RAM blocks, DSPs, PLLs, and others. The tool Vivado EDA has been used for the synthesis and implementation process, adopting strategies oriented to the optimization of the performance (of timing and power, notably the synthesis strategy denoted as Flow_PerfOptimized_high and the implementation strategy denoted as Performance_ExtraTimingOpt, respectively). Table 3 reports the final results of this activity.

Regarding the synthesis results on standard-cell technology, in this case, the Design Compiler EDA by Synopsys was used, and the target technology was the one proposed by the EPI project, i.e., the H300 BASE SVT C8 of the 7 nm TSMC process CLN07FF41001 SVT, and released by ARM as part of the package of logic products named Artisan 7 nm TSMC CLN07FF41001. The operating conditions and the technology corner case used in the synthesis were 0.90 V for the voltage supply, 125 °C for temperature, and slow process. Table 4 summarizes the final post-synthesis results on the 7 nm standard-cell technology by ARM, in which the area data are expressed in Gate Equivalent (GE), being 1 GE corresponding to the area of the two-inputs NAND gate of the ARM Artisan 7 nm technology with the smallest area, i.e., 0.0768 μ^2^.

### 3.2. RNG Assessment

Several suites exist to evaluate the randomness of an RNG; over the years, researchers have proposed different statistical methods and tests to assess the output sequences of both hardware and software implementations of RNG. The first and most notorious was the Diehard, which has been updated and improved to become the suite Dieharder [22]. Other suites of the test have been proposed in recent years, including the following:

- The ENT suite [23], for example used in [24,25];
- The batteries of tests PractRand [26], employed in [27,28], and TestU01 [29], whose usage is reported in [27,28,30,31,32,33]: both essentially represent an enhancement of Diehard(er) suite, because they include some improvements such as the possibility of setting the parameters of some of the offered statical tests (feature not offered by Diehard(er));
- The NIST Statistical Test Suite (STS) [34], used in [24,25,30];
- The battery of tests described in [35], in order to meet the requirements of the BSI standards AIS 20 [36], for DRBGs and AIS 31 [37], for TRNGs, whose usage is documented by [38,39];

A review on the available statistical tests available can be found in [40]. In case of cryptographic applications, the most indicated statistical suites, as confirmed by [38,41], are the ones offered by the standardization organizations, i.e., NIST and BSI. Hence, their usage is indicated to evaluate two fundamental aspects for implementations of RNGs: the entropy and the randomness of bitstreams. The goal of the former metric concerns the quantification of how many bits of entropy an RNG module can generate; the latter is to evaluate how much the sequences of random bits generated by a real RNG differ from the sequences produced by an ideal RNG. In this work, we adopted both the NIST and BSI suites for the assessment of our RNG.

The BSI suite is made available as a Java-based tool that can be downloaded at [42], and it includes a battery of statistical tests divided into two distinct procedures: procedure A, comprising the tests *T*0 and *T*1 through *T*5, which is aimed to evaluate the randomness of sequences of bits, and procedure B, comprising the tests *T*6 to *T*8, whose purpose is to calculate the entropy level of such sequences [35], which is an update of both [43,44], gives mathematical references of the tests, and describes the procedures to be followed for both TRNGs (AIS 31) and DRBGs (AIS 20).

The NIST offers two distinct suites for the two distinct characteristics to be analyzed: the Entropy Assessment (EA) suite, illustrated in the standard NIST SP 800-90B [45], and the already mentioned STS described in the standard NIST SP 800-22 [34]. The former is required for the evaluation of the entropy and is available as a collection of software applications written in C++ language that can be downloaded through a git repository at [46]. The latter can be used for testing the randomness of sequences and is made available as a C software application, whose most recent version is 2.1.2, and it can be downloaded from the NIST website at [47].

#### 3.2.1. Entropy Evaluation

The entropy evaluation of an RNG becomes relevant when it is used to generate seeds for PRNGs or CSPRNGs, as in the case of the entropy source we used in this work to supply the DRBG. By evaluating the level of entropy, it is then possible to determine if the RNG module can satisfy the requirements of the minimal amount of bits of entropy required by the DRBG mechanism intended to use such seeds as the ones specified by the NIST in [9]. The EA suite for the evaluation of the entropy essentially counts two steps: a preliminary entropy assessment routine for the calculation of the initial entropy $H_{I}$ and a successive entropy assessment routine that estimates the entropy per row $H_{R}$, and the entropy per column $H_{C}$, of a 1000 × 1000 matrix of random samples. The routine outputs the final value of entropy *H*, which is calculated as the minimum between $H_{I}$, $H_{R}$, and $H_{C}$:

$$H=min(H_{I},H_{R},H_{C}).$$

The required samples have been acquired from the entropy source module exploiting the dedicated debug probe signal in Figure 1, and the estimated entropy is as follows:

- 7.888 bits of entropy per byte, corresponding to 0.986 bit of entropy per bit, estimated with the NIST EA suite;
- 7.999 bits of entropy per byte, corresponding to 0.999 bit of entropy per bit, estimated with the BSI suite.

The difference among values generated by the two statistical suites may find an explanation in the fact that, as reported and highlighted by [48,49], the estimators of NIST SP 800-90B (i.e., NIST EA) are subject to systematic underestimates of entropy. Anyway, also in the worst case (the NIST suite one), the obtained level of entropy per bit allow achieving an overall entropy of 0.986×512=504.832 bits for the seeds generated by the entropy source module of the RNG engine (Section 2.1, Figure 1) and used as inputs of DRBG mechanism of the same engine, and such a value largely satisfies the requirement of entropy of at least 256 bits, according to NIST specifications in [9].

#### 3.2.2. Randomness Tests

For the randomness evaluation, both the NIST STS and the BSI suite are formulated to test a specific null hypothesis $H_{0}$ (i.e., the sequence being tested is random), and an alternative hypothesis $H_{a}$ (i.e., the sequence being tested is not random). Such hypotheses are defined to address all the possible cases that can occur in the generation–conclusion procedure when testing the randomness of a bitstream, as reported by Table 5. The probability of Type I error, i.e., the event that a random generator is declared being non-random, is defined as α, called significance level, while the probability of Type II error, i.e., the event that a non-random generator is declared being random, is denoted by β [34]. The author of [34] defines a range of admitted values for α and two metrics to evaluate the randomness of a sequence under test. Such metrics rely on the calculation of the so-called *p*-value, which is defined as the probability that a perfect random number generator would have produced a sequence less random than the tested sequence. Then, the conclusion on the randomness of a sequence can be determined basing on the relation between the *p*-value and α: if *p*-value ≥α, then the sequence can be considered random with a confidence of (1−α)·100%; otherwise, if *p*-value < α, the sequence can considered as non-random with a confidence of (1−α)·100%. For cryptographic applications, α is typically chosen in the range (0.001–0.01). In addition, also, the number of sequences to be tested is regulated by the indication that if we defined *k* as the number of *n*-bit sequences to be tested, then k≥1/α: i.e., for α=0.01, at least 100 sequences have to be tested, while for α=0.001, at least 1000 have to be tesetd. Based on this, each of the 15 statistical tests composing the NIST STS operates as follows:

- The *p*-value of each sequence is calculated, discarding the sequences for which *p*-value < α;
- The ratio between the number of sequences that passed the test (i.e., the one for *p*-value ≥α) and the total number of tested sequences (i.e., *k*) is computed, and it is labeled as PRoportion (PR);
- The *p*-value of sequences that passed the test are distributed in the range [0,1) by splitting it into 10 equal sub-intervals, and the uniformity of the distribution of *p*-value is calculated: basing on the chi-square (chi-squared or $\chi^{2}$) function, the uniformity of distribution is determined by computing a figure that can be considered as a *p*-value of *p*-value (PoP);

Then, all of the results above are reported for each of the tests, and a final and global conclusion on the randomness of the generator that produced the tested sequences can be derived, by declaring it as random, with a confidence of (1−α)·100%, if:

- For each test, PR lies in the confidence interval defined as $\left(1-\alpha \right)\pm 3\sqrt{\frac{\alpha (1-\alpha )}{k}}$;
- For each test, PoP ≥ 0.0001 (i.e., the *p*-values of sequences that passed the test are uniformly distributed).

**Figure 7:**
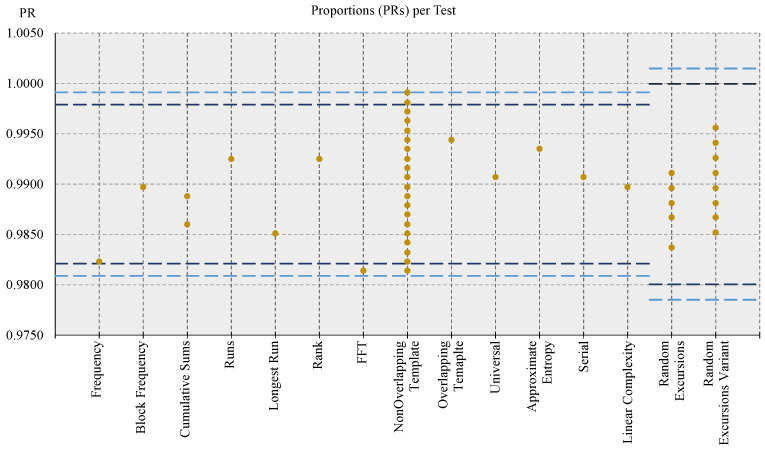
Graph of NIST STS PR metric results for tested DRBG sequences. The dark golden points represent the PR values for each test, or sub-test, and the blue dashed lines represent the boundaries of the confidence interval(s): it the PR value lies outside the confidence interval(s), the test is failed: three (six) failing tests are tolerated for the widest (narrowest) confidence interval.

**Figure 8:**
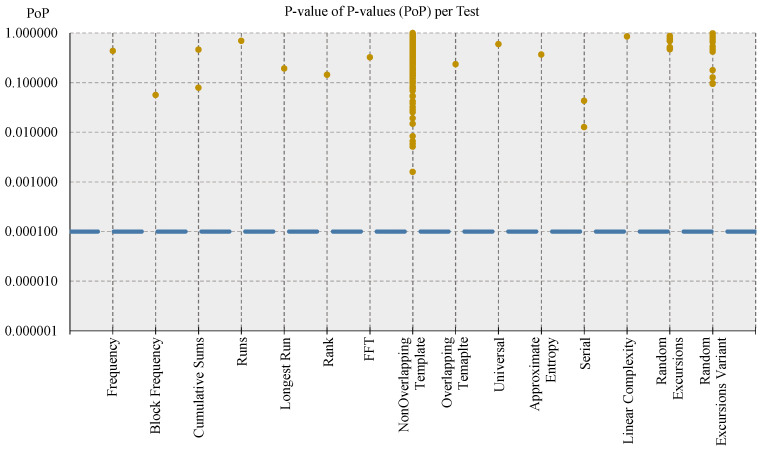
Graph NIST STS PoP metric results for tested DRBG sequences. The dark golden points represent the PoP values for each test, and the light blue dashed line traces the threshold for the pass–fail criterion: if the PoP value is greater than (or equal to) threshold, then the test is passed; otherwise, it is failed. The vertical axis uses the logarithmic scale.

Regarding the evaluation of the randomness of the DRBG module (and hence of the overall CSPRNG unit corresponding to the RNG engine), its output sequences have been tested with both procedure A of the BSI suite (notably the battery of tests *T*0 and *T*1 through *T*5) and the Fast NIST STS, using the configuration parameters for NIST STS indicated by the BSI in [35] (α = 0.01, *k* = 1073, *n* = 1,000,000, block length of Block Frequency test *M* = 20,000, template and block length for Non-Overlapping Template and Overlapping Template tests *m* = 10, block lengths of Approximate Entropy, Serial, and Linear Complexity tests, respectively, *m* = 8, *m* = 16, and *M* = 1000). In both cases, all tests passed, confirming the indistinguishability of the bistreams generated by the RNG engine from the ones produced by an ideal RNG, in particular, with a confidence of 99%, according to the specifications of SP 800-22 document. Figure 7 reports a representation of the results for the proportion metric, while Figure 8 reports the one for the uniformity metric.

**Figure 9:**
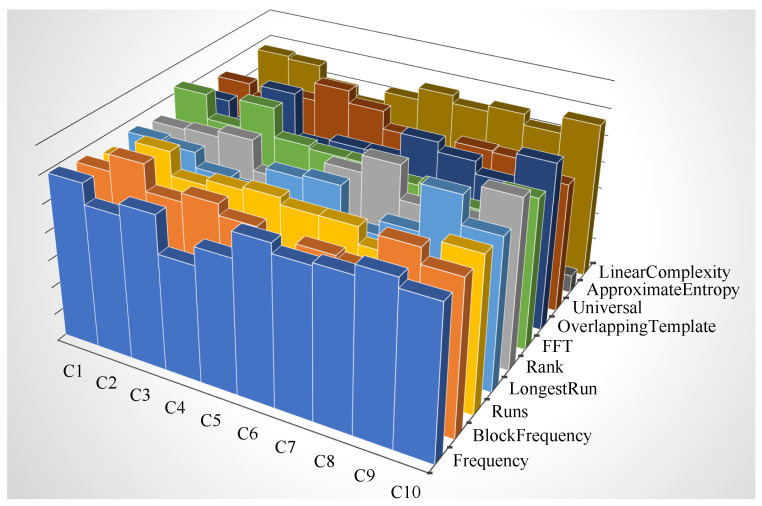
Histograms of *p*-values distributions from NIST STS tests. The three-dimensional histograms of the distribution of *p*-values are reported only for single-experiment tests of NIST STS and, according to the results of PoP metric (Figure 8), their uniformity can be noted.

Referring to Figure 7, the dark golden dots represent the PR values for each test, while the blue dashed lines represent the upper and lower limits of the confidence interval used to determine if a sequence passed a test: the light blue ones delimit the confidence interval deriving from the original formula of NIST SP 800-22, i.e., the one with the value 3 for the constant, and the dark blue ones delimit the corresponding confidence interval using a value of 2.6 for the constant in the formula, according to [50]. Interpreting the graphical representation of results, on one hand, multiple dark golden dots (i.e., PR values) on the same vertical line means that the corresponding test is composed of multiple sub-tests, and the outcome of each of them is reported; on the other one, it can be noted also that some of the tested sequences failed some of the test (i.e., the associated dark golden dot lies outside the confidence interval). This must not be interpreted as a failure of the NIST STS testing procedure, because, according to [50], a certain number of failing tests can be tolerated without affecting the outcome of the experiment. The number of tolerated failing tests is three (or six) when referring to a confidence interval computed using a constant value of 3 (or 2.6). In the former case, no test (0) exceeded the limit of the confidence interval related to the constant value of 3, while in the latter case, only five tests lie outside the confidence interval related to the constant value of 2.6; hence, the final result is that the tested sequences successfully passed the NIST STS tests, according to the proportion metric. The same graphical representation approach applies also to Figure 8, showing that the PoP values (dark golden dots) of all tests are greater than the threshold of 0.0001 (dashed light blue line) used for the pass–fail criterium, indicating the distributions of *p*-values were shown to be uniform, as illustrated by Figure 9.

As an additional step, also, the bitstreams of the entropy source module were tested with the NIST STS software, and also, in this case, the sequences passed all tests, confirming that such a unit can be used also as a stand-alone TRNG, being already equipped with hardware resources to the health test of the raw binary stream.

## 4. Comparison to the State of the Art

To make a comparison with the state of the art, ref. [39] offers a good reference point, because it offers a review of several implementations of TRNG on FPGA technologies, by using the AIS 31 suite for the estimation of entropy. The FPGA analyzed in [39] are the Intel Cyclone V [51], the Xilinx Spartan 6 [52], and the Microsemi SmartFusion 2 [53], respectively manufactured on technology nodes at 28, 45, and 65 nm, and the characteristics reported for the TRNG involve both the entropy per bit and the entropy rate, which were calculated as the entropy per bit multiplied by the bit rate, according to the main target application of these modules to generate seeds with a certain amount of bits of entropy (as expressed above). Even if a TRNG, or an entropy source module, can produce an elevated level of entropy per bit, it may anyway not be suitable for some applications in case the generation rate is too low, or, in other words, the latency it features is too high, when compared to the interval time that can be considered acceptable for the accumulation of a certain amount of entropy bits. Table 6 includes data extracted from [39] that refer to the corresponding publications in the literature.

The results in Table 6 show that the entropy source module integrated into the RNG engine and implemented on the VCU128 demo board offers a level of entropy equal to the maximum one provided also by other solutions (and very close to the maximum theoretical one of 1.000), and that it features also the best entropy rate, which is about 8.5 times greater than the best one of other FPGA implementation. In addition, if assuming the entropy estimated by the NIST EA, i.e., 0.986, the corresponding bit rate was 2080×0.986=2050.88 Mbit/s, which is much greater than the best one of the counterparts that are 244.755 Mbit/s. This was confirmed also by the work presented in [6] in which it presented the implementation of a TRNG on an Intel Stratix IV FPGA [64], and on which basis the entropy source module was integrated into the RNG engine: in that work, the measured entropy was of 0.995, and the bit rate was 400 Mbit/s, thus leading to an entropy rate of 398 Mbit/s.

Please note that the largely better performance in terms of throughput is due to both the FPGA technology being significantly newer and the amount of resources used (e.g., the more resources you use in parallel, the larger throughput you can achieve). We reported the throughput as a figure of merit, but we did not center our analysis on that, knowing that it would not be fair. As a further and general metric to evaluate the entropy estimation, as suggested by the authors of [65], the minimum value of entropy per bit should satisfy the requirement of 0.910, as specified by [35] in the form of the Shannon entropy of 0.997. The Shannon entropy ($H_{S}$) can be calculated from the value of minimum entropy (or min-entropy, $H_{min}$, i.e., the one computed by the NIST EA and BSI suite), by the following formula:

$$H_{S}=-2^{-H_{min}}\cdot \log_{2}\left(2^{-H_{min}}\right)-\left(1-2^{-H_{min}}\right)\cdot \log_{2}(1-2^{-H_{min}}).$$

To derive the equation above, we relied on the following assumptions: approximation of Shannon entropy as the sum of probabilities [36], and the fact that the random bit can assume only two values (0 and 1). These are also specified and exploited in [65].

Using only the expression above, the minimum value of $H_{S}=0.997$ corresponds to the value of $H_{min}$ equal to 0.910. The author of [35] specifies such a value as one of the requirements that an RNG must respect to be compliant with the class-defined *PTG.2*, the one collecting PTRNG whose target is the generation of cryptographic keys, seeds, and random padding bits. Hence, the corresponding value (the effective values of $H_{S}$ are 0.99993141 and 0.99999965 for $H_{min}$ values of 0.986 (NIST EA) and 0.999 (procedure B of BSI suite), respectively, that can be rounded both to the value 1.000 when using 4 significant digits for representing those numbers) of $H_{S}$ is 1.000 for both the min-entropy values of 0.986 and 0.999, enabling the entropy source module to be an RNG of class *PTG.2*. Successively, procedure A of the BSI suite was applied to the random sequences of the entropy source module, according to [35], and all tests passed, confirming the compliance to the European requirements for TRNG expressed by the AIS 31 standard.

## 5. Conclusions

In this work, we implemented a high-entropy and high-throughput RNG, which demonstrated to be portable in different technologies. We validated the correct implementation of the RNG engine as a CSPRNG, and we verified that it satisfies the security requirements for cryptographic applications, because as confirmed by the results, once seeds with appropriate entropy levels are used, it can generate sequences whose randomness cannot be distinguished from the one of an ideal random generator, with the confidence of 99%. As such characteristics are depending only on the deterministic part of the RNG engine, it will be maintained also for the EPI chip on the 7 nm standard-cell technology. In addition, the measured level of entropy contributed to proving the portability of the digital entropy source module, showing also characteristics in terms of entropy rate, which outperforms the other main solutions in the field of digital TRNGs. The extracted value of 1.000 for the Shannon entropy per bit makes it a candidate for the PTG.2 class of AIS 31 standard, i.e., a (physical) TRNG for the generation also of certified and highly-qualified cryptographic keys.

## Figures and Tables

**Table 1:** Comparison of design strategies for digital entropy source modules. The approach based on TERO is illustrated in [11,15,16,17], the one based on Meta-RO is described in [12], while some examples of FiROs and GaROs can be found in [13], and the one employing FiGaRO is presented in [13].

Design Strategy	Physical Phenomena Generating Entropy	Main Characteristics
TERO	Latches oscillatory metastability	Low throughputs, large dependence on placement of logic cells
Meta-RO	Analogue metastability of inverter gates	PLL required, dependence on placement of logic cells
FiRO	Jitter and metastability	Good independence from placing
GaRO	Jitter and metastability	Good independence from placing
FiGaRO	Jitter and metastability	Independence from placing, higher entropy and robustness respect to single FiRO and GaRO

**Table 2:** Truth table of *NAND* gate.

*B*	*A*	C=B·A	$Y=\bar{C}=\bar{B\cdot A}$
0	0	0	1
0	1	0	1
1	0	0	1
1	1	1	0

**Table 3:** Implementation results on FPGA VU37P. The percentage data between round brackets refer to the relative utilization of the corresponding entity with respect to the total of resources offered by the FPGA device.

Entity	Frequency [MHz]	CLB (162,960)	CLB LUTs (1,303,680)	CLB Registers (2,607,360)
RNG engine	260	2151	9842	7121
Entropy Source	260	384	1567	2137
DRBG	260	1528	7327	3685

**Table 4:** Post-synthesis results for the RNG engine on 7 nm technology. Frequency data are expressed in GHz, while area data are expressed in kGE.

Entity	Frequency [GHz]	Area [kGE]
RNG engine	4.325	127.16
Entropy Source	4.325	69.29
DRBG	4.325	46.51

**Table 5:** Possible outcomes of randomness evaluation procedure.

True Situation	Conclusion	
	Data Are Random (Accept H0)	Data Are Not Random (Accept Ha)
Data are random (H0 is true)	No error	Type I error
Data are not random (Ha is true)	Type II error	No error

**Table 6:** Comparison between FPGA implementations of TRNG. Data reported in this table have been extracted from the results of this works and from [39]. In case multiple citations are present, it indicates that the analyzed TRNG implementation was built merging the contributions from each of the works documented in the corresponding citation.

Implementation	FPGA	Bit Rate [Mbit/s]	Entropy per Bit (from BSI Suite)	Entropy Rate [Mbit/s]
This work	Virtex Ultrascale+ VU37P	2080	0.999	2077.92
[54]	Spartan 6	0.0042	0.999	0.004
	Cyclone V	0.0027	0.990	0.003
	SmartFusion 2	0.014	0.980	0.013
[55]	Spartan 6	0.54	0.999	0.539
	Cyclone V	1.44	0.999	1.438
	SmartFusion 2	0.328	0.999	0.327
[56,57,58]	Spartan 6	2.57	0.999	2.567
	Cyclone V	2.2	0.999	2.197
	SmartFusion 2	3.62	0.999	3.616
[59,60]	Spartan 6	0.44	0.981	0.431
	Cyclone V	0.6	0.986	0.592
	SmartFusion 2	0.37	0.921	0.340
[11,61]	Spartan 6	0.625	0.999	0.624
	Cyclone V	1	0.987	0.985
	SmartFusion 2	1	0.999	0.999
[62,63]	Spartan 6	154	0.998	154.121
	Cyclone V	245	0.999	244.755
	SmartFusion 2	188	0.999	188.522

## Data Availability

Not applicable.
